# Clemastine protects against sepsis-induced myocardial injury *in vivo* and *in vitro*

**DOI:** 10.1080/21655979.2022.2047256

**Published:** 2022-03-11

**Authors:** Xiaowan Wang, Di Xie, Hui Dai, Jiawei Ye, Yuqi Liu, Aihua Fei

**Affiliations:** a Department of Emergency, Xinhua Hospital Affiliated to Shanghai Jiaotong University School of Medicine, Shanghai, China; b Department of General Practice, Xinhua Hospital Affiliated to Shanghai Jiaotong University School of Medicine, Shanghai, China

**Keywords:** Sepsis-induced myocardial dysfunction, clemastine, autophagy, apoptosis, mitochondrial damage, histamine receptor blocker

## Abstract

Sepsis-induced myocardial dysfunction (SIMD) is associated with high morbidity and mortality rates; however, it lacks targeted therapies. Modulating cardiomyocyte autophagy maintains intracellular homeostasis during SIMD. Clemastine, a histamine receptor inhibitor, promotes autophagy and other effective biological functions. Nevertheless, the effect of clemastine on SIMD remains unclear. This study aimed to explore the underlying mechanism of clemastine in cardiomyocyte injury in cecum ligation and perforation (CLP)-induced rats and lipopolysaccharide (LPS)-stimulated H9c2 cells. Clemastine (10 mg/kg, 30 mg/kg, and 50 mg/kg) was intraperitoneally injected after 30 min of CLP surgery. Serum cTnI levels and the 7-day survival rate were evaluated. Echocardiograms and H&E staining were used to evaluate cardiac function and structure. TEM was used to detect the mitochondrial ultrastructure and autophagosomes. Clemastine significantly improved the survival rate and reduced cTnI production in serum. Clemastine ameliorated cellular apoptosis, improved mitochondrial ultrastructure both *in vivo* and *in vitro*, increased ATP content, decreased dynamin-related protein 1 (DRP1) expression, and decreased mitochondrial ROS levels. Additionally, clemastine treatment increased autophagosome concentration, LC3II/LC3I rate, and Beclin 1 expression. However, 3-methyladenine (3-MA), an autophagy inhibitor, could abolish the effect of clemastine on alleviating myocardial apoptosis. In conclusion, clemastine protected against cardiac structure destruction and function dysfunction, mitochondrial damage, apoptosis, and autophagy *in vivo* and *in vitro*. Moreover, clemastine attenuated myocardial apoptosis by promoting autophagy. This study provides a novel favorable perspective for SIMD therapy.

## Graphical Abstract

**Figure:**
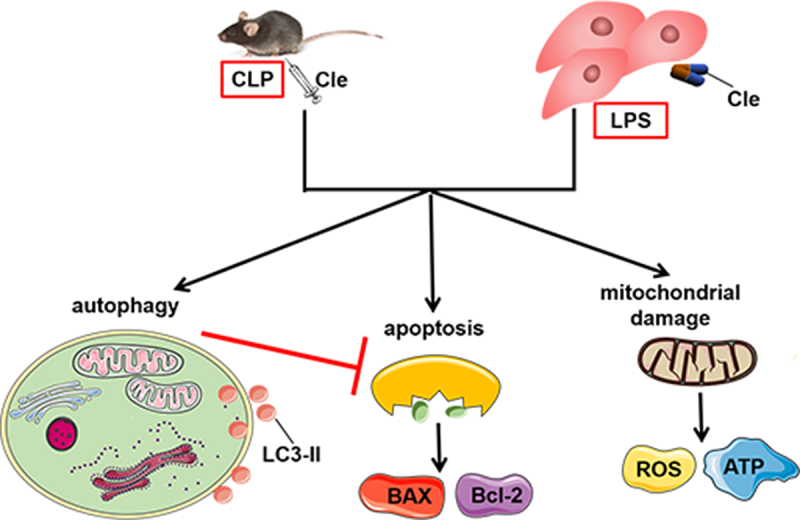

## 1. Introduction

Sepsis is a common and fatal disease in emergency departments. Approximately 48.9 million cases of sepsis and 11 million cases of sepsis-related deaths have been reported globally, and these estimates are doubling every year [1,2]. Sepsis-related organ dysfunctions mostly affect the heart. The incidence of sepsis-induced myocardial dysfunction (SIMD) is 64%, and the fatality rate is 70–90% [3,4]. Such high morbidity and mortality rates impose an enormous financial burden on both healthcare systems and society [5]. However, current treatments mainly focus on supportive care with early fluid resuscitation and antibiotics [6,7]. Further, there is no targeted intervention in sepsis, specifically for myocardial dysfunction. Therefore, effective therapies for SIMD are urgently needed and extremely significant.

Cardiomyocytes have a limited ability to differentiate and regenerate. Autophagy provides energy and promotes material circulation and cellular self-renewal by degrading misfolded proteins and dysfunctional or aging organelles [8,9]. Therefore, myocardial autophagy is crucial for maintaining cardiac function and vitality. Basal levels of autophagy can maintain normal physiological balance of cardiomyocytes; however, autophagy can significantly increase as an adaptive response to stress in the pathological state [10]. Many studies have illustrated that the pathogenesis of cardiac anomalies in sepsis is highly correlated with autophagy [11,12]. Modulation of autophagy exhibits protective effect pharmacologically in cecum ligation and perforation (CLP)-constructed sepsis model and lipopolysaccharide (LPS)-induced cardiomyocytes, suggesting that autophagy is an adaptive response in SIMD [13,14]. Mechanistically, modulating cardiomyocyte autophagy degrades misfolded proteins and damaged cellular components to maintain intracellular homeostasis during SIMD [15]. Further, previous studies have revealed that promoting autophagy alleviates myocardial apoptosis, improves cardiac dysfunction, and increases the survival rate of septic rats [16,17]. A recent study showed that cardiac autophagy changes dynamically during the progression of sepsis, and autophagy is insufficient and maladaptive in the later stages of sepsis, leading to serious consequences [18]. Therefore, maintaining adequate autophagy levels is a challenging problem, and the identification of specifically targeted autophagy agonists provides a new opportunity for treatment of SIMD.

Clemastine fumarate (Cle), a second-generation H1 receptor blocker, is widely used in clinical practice to treat allergic diseases. Studies have reported that clemastine exhibits many potential pharmacological activities, including the promotion of autophagy [19,20] and anti-apoptosis [21]. Moreover, a previous study revealed that clemastine attenuates myocardial apoptosis to protect against myocardial ischemia-reperfusion injury [22]. However, no study has employed a sepsis model to elucidate the role and underlying mechanism of clemastine in SIMD.

Given that the treatment for SIMD is not ideal and clemastine displays multiple effective biological functions, such as improving autophagy and anti-apoptosis, we conducted this study to examine the possible effect of clemastine on SIMD and to elucidate the underlying mechanism. This study was designed using a CLP-induced rat model and an LPS-induced H9c2 cardiomyocyte model. By verifying the effect of clemastine on cardiac function, apoptosis, mitochondrial damage and dysfunction, and autophagy, our findings may provide novel insights and pave the way for strategic treatment of humans with SIMD.

## 2. Materials and methods

### 2.1. Experimental animals

Adult male Sprague Dawley rats (weight: 220–250 g) were obtained from the Laboratory Animal Center of Xinhua Hospital (Shanghai, China). The rats were housed in a specific pathogen-free environment under regular 12 h/12 h light-dark cycles at a temperature of 24 ± 2°C and humidity of 50–60%. Prior to the experiments, all animals were allowed a week to fully adapt to the environment. All animal procedures strictly adhered to the rules and requirements of the Laboratory Animal Ethical and Welfare Committee of Xin Hua Hospital affiliated to Shanghai Jiao Tong University School of Medicine. The animal experiments were approved by Ethics Committee of Xin Hua Hospital Affiliated to Shanghai Jiao Tong University School of Medicine (XHEC-F-2018-038).

### 2.2. CLP and experimental protocols

Following hair removal from the abdomen, a 1.5-cm incision was made along the midline of the abdomen to fully expose the cecum. Subsequently, the cecum was ligated and perforated using a sterile 22-gauge needle. Finally, the abdominal musculature and skin were closed. The sham group underwent only laparotomy and cecum exposure as previously described [23].

Three different doses of Cle (10 mg/kg, 30 mg/kg, and 50 mg/kg) (Meilunbio, Dalian, China) were dissolved in saline solution and administered after 30 min of CLP surgery via intraperitoneal injection. 15 mg/kg 3-methyladenine (3-MA) (Aladdin Regents, Shanghai, China) was administered to rats in the CLP + Cle group immediately after CLP surgery via intraperitoneal injection as previously described [24]. Equal volumes of normal saline (NS) served as controls.

After adaptation to the housed environment for one week, rats were randomly grouped by the random number table method as follows:1) To assess whether clemastine improves survival rate and decreases serum cTnI levels in septic rats and to observe the impact of different doses clemastine, rats were randomly grouped as follows: (1) Sham + NS; (2) CLP + NS; (3) CLP + Cle-10; (4) CLP + Cle-30; and (5) CLP + Cle-50; 2) After determining the most suitable dose, to assess the effect of clemastine treatment on echocardiography, hematoxylin-eosin (H&E) saining, apoptosis, autophagy, and mitochondrial damage, rats were randomized into another three groups as follows: (1) Sham + NS; (2) CLP + NS; and (3) CLP + Cle groups; 3) To assess whether clemastine functions in myocardial apoptosis via promoting autophagy, the remaining rats were randomized into another four groups as follows: (1) Sham + NS; (2) CLP + NS; (3) CLP + Cle; and (4) CLP + Cle + 3-MA groups. During the experiment, data collection and analysis were conducted blindly, and the experimenters were blinded to the groups.

### 2.3. Cell culture and treatment

H9c2 cardiomyocytes were obtained from the National Collection of Authenticated Cell Cultures (Shanghai, China). H9c2 cells were grown in Dulbecco’s Modified Eagle Medium (DMEM) containing 10% fetal bovine serum (Hyclone, South America), 1% streptomycin, and 1% penicillin (Yeasen, China). When cells reached 80% confluence, they were passaged with 0.25% trypsin (Gibco, CA, USA), and subsequently, they were seeded onto six-well plates for western blotting and onto twelve-well plates for Terminal deoxynucleotidyl transferase dUTP nick end labeling (TUNEL) staining. The cells were randomly assigned into three groups, control group (without LPS or clemastine), LPS group (10 µg/mL, Escherichia coli O55:B5, Sigma-Aldrich, St. Louis, MO, USA), and LPS + Cle group (10 µg/mL LPS and 1.25 ug/mL clemastine).

### 2.4. Echocardiography

After 24 hours of CLP surgery, rats were gradually sedated with inhaled isoflurane (1.5%–2%), and they were exposed to transthoracic echocardiography using Vevo 2100 (VisualSonics, Toronto, Canada). Subsequently, left ventricular ejection fraction (LVEF), left ventricular fraction shortening (LVFS), left ventricular end-systolic diameter (LVESD), and left ventricular end-diastolic diameter (LVEDD) values were scrutinized and recorded for cardiac function analysis in rats.

### 2.5. Histological examinations

Left ventricle specimens of the heart were fully fixed with 4% paraformaldehyde for 12 h, embedded into wax stones, and cut into sections. After xylene transparency, gradient dehydration with ethanol, hematoxylin staining, eosin staining, alcohol dehydration transparency, and resin sealing, the tissue slides were observed under a microscope.

### 2.6. TUNEL staining

Paraffin sections were prepared as described above, and a commercial TUNEL detection kit (Beyotime, Shanghai, China) was employed to detect apoptotic cells following manufacturer’s instructions.

### 2.7. Paraffin section immunohistochemistry

Paraffin sections were prepared according to the procedures mentioned above. Subsequently, the sections were dewaxed with xylene, repaired with citric acid antigen, blocked with serum, and incubated with SOD2 antibody (#13,141, CST). Images were visualized under a microscope.

### 2.8. Transmission electron microscopy (TEM)

The left ventricular wall of the heart and H9c2 cells were harvested and fixed with 2.5% glutaraldehyde (Servicebio, Wuhan, China). Subsequently, they were dehydrated with alcohol, embedded in epoxy resin, and divided into 70-nm sections using an ultrathin microtome. Finally, the images were observed using an electron microscope.

### 2.9. Determination of cardiac troponin I (cTnI) levels in serum

The collected whole blood was centrifuged at 3000 rpm for 10 min to obtain serum. Subsequently, ELISA kits for cTnI (Elabscience, Wuhan, China) were used to detect cTnI levels following manufacturer’s instructions.

### 2.10. Measurement of cardiac adenosine triphosphate (ATP) content

The ATP assay kit (Nanjing Jiancheng Bioengineering Institute, China) was employed to detect ATP content in the fresh left ventricle of the heart following manufacturer’s instructions.

### 2.11. Dihydroethidium (DHE) staining

ROS levels were evaluated using DHE dye (Wako Chemical, Osaka, Japan). The left ventricle of the heart was cut into sections and incubated with DHE staining solution for 20 min. Subsequently, images were observed using a fluorescence microscope.

### 2.12. Western blots

The left ventricle of the heart and cultured H9c2 cells were fully lysed to obtain the protein. Samples were separated and then transferred to a 0.45-μm PVDF membrane (Millipore, MA, USA) with 8%-12.5% SDS-PAGE rapid electrophoresis buffer and rapid transfer buffer. Subsequently, the membranes were fully incubated with specific antibodies, LC3 (ab192890, Abcam), Beclin 1 (3495 T, CST), Bcl-2 (#15,071, CST), BAX (A20227, Abclonal), DRP1 (#8570, CST), and GAPDH (ab8245, Abcam). The membranes were further incubated with goat anti-mouse antibodies (#7076, CST) or goat anti-rabbit antibodies (#7074, CST), and they were imaged with an Amersham Imager 600.

### 2.13. Statistical analysis

All experimental data were evaluated using SPSS 20.0 (SPSS, Inc., Chicago, USA). The distribution values are presented as mean ± standard error. Univariate analysis of variance was used to estimate different groups of univariate data. Differences were considered statistically significant at *p* < 0.05.

## 3. Results

Several methods were employed to explore the protective mechanisms of clemastine against sepsis-induced myocardial dysfunction *in vivo* and *in vitro*. Our data showed that clemastine treatment significantly improved sepsis-induced cardiac dysfunction via mitochondrial protection, apoptosis inhibition, and autophagy improvement both *in vivo* and *in vitro*. Moreover, clemastine ameliorated myocardial apoptosis in CLP-induced cardiac dysfunction via autophagy.

### 3.1 Clemastine attenuates CLP-induced cardiac dysfunction

**Figure 1.**
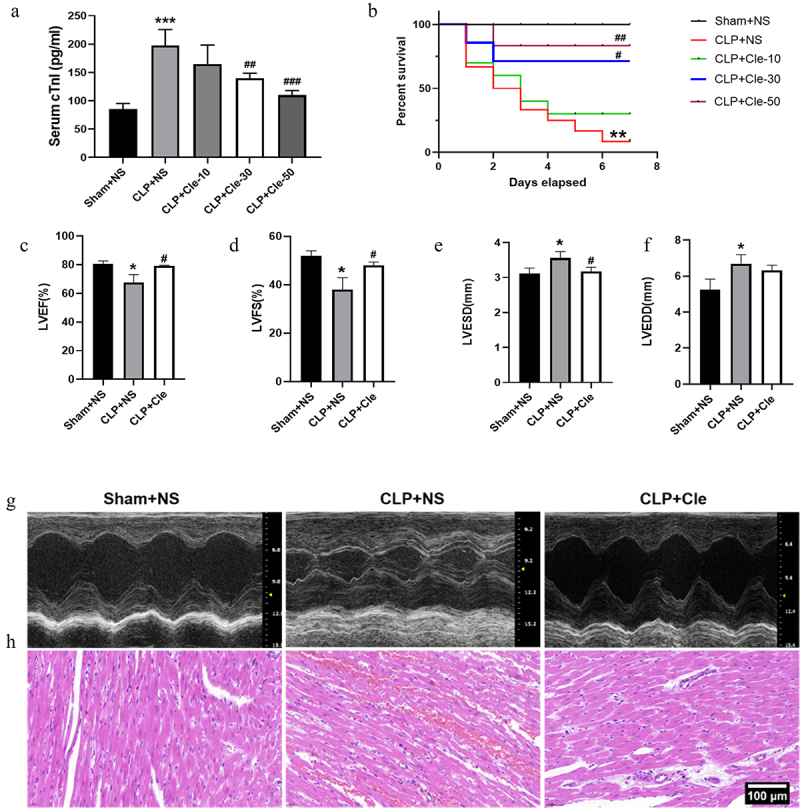
Clemastine attenuates CLP-induced cardiac dysfunction. The effect of different doses of clemastine was assessed via serum cTnI level and the 7-day survival rate. The structure and function of the heart were evaluated using echocardiography and H&E staining. (a) cTnI (n = 12); (b) 7-day survival rate (n = 12); (c-f) Quantification of LVEF, LVFS, LVESD, LVEDD via echocardiography (n = 3); (g) Representative echocardiographic images (n = 3); (h) Myocardium H&E staining (n = 3). Data are expressed as mean ± SD, *P < 0.05 vs Sham+NS, **P < 0.01 vs Sham+NS, ***P < 0.001 vs Sham+NS, ^#^ *P* < 0.05 vs CLP+NS, ^##^ *P* < 0.01 vs CLP+NS, ^###^ *P* < 0.001 vs CLP+NS.

Rats were exposed to CLP surgery to establish a sepsis model, and they were administered clemastine at three different doses (10, 30, and 50 mg/kg). Subsequently, serum cTnI levels were evaluated, and the 7-day survival rate was monitored and calculated. Overall, clemastine significantly improved survival and decreased serum cTnI levels. Serum cTnI levels were significantly downregulated in CLP+Cle-50 and CLP+Cle-30 groups as compared to those in CLP group (Figure 1a). Consistently, the survival rate was significantly upregulated in CLP+Cle-30 and CLP+Cle-50 groups as compared to that in CLP group (Figure 1b). Nevertheless, 10 mg/kg clemastine had little effect on serum cTnI level and the 7-day survival rate in CLP-induced rats. Therefore, 30 mg/kg clemastine was used for subsequent *in vivo* experiments.

Echocardiography and H&E staining were performed to evaluate the cardiac structure and function of rats. After 24 h of CLP surgery, LVEF and LVFS values significantly decreased and LVESD significantly increased in CLP+NS rats as compared to those in sham-operated rats. Interestingly, clemastine treatment significantly increased LVEF and LVFS and decreased LVESD in rats with CLP surgery (Figure 1c-e). However, no significant change was observed in LVEDD (figure 1f). H&E staining revealed disordered and broken myocardial fibers, with significant edema, red blood cell exudation, and inflammatory cell infiltration in myocardial tissues of CLP+NS group. Interestingly, these deteriorations were attenuated in CLP+Cle group (Figure 1h).

### 3.2 Clemastine ameliorates cellular apoptosis in rat heart and H9c2 cells

**Figure 2.**
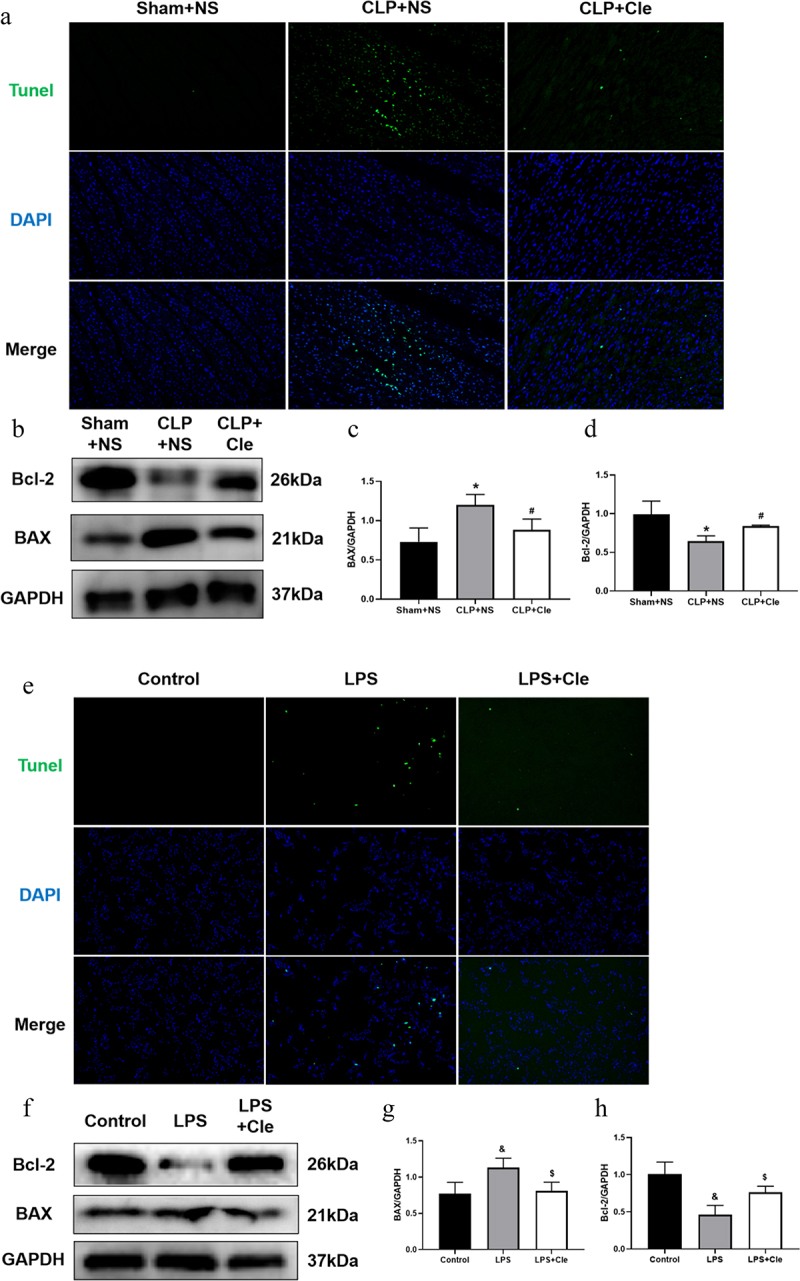
Clemastine ameliorates cellular apoptosis in rat hearts and H9c2 cells. (a) Representative images depicting TUNEL-positive nuclei (200X) from respective rat groups; (b) Representative gel blots depicting the levels of BAX and Bcl-2 *in vivo* (GAPDH was used as the internal reference protein); (c) BAX/GAPDH levels and (d) Bcl-2/GAPDH levels (n = 3). (e) Representative images depicting TUNEL-positive nuclei from respective H9c2 cells; (f) Representative gel blots depicting the levels of BAX and Bcl-2 *in vitro*; (g) BAX/GAPDH levels; (h) Bcl-2/GAPDH levels (n = 3). *P < 0.05 vs Sham+NS, ^#^*P* < 0.05 vs CLP+NS; ^&^*P* < 0.05 vs Control, ^$^*P* < 0.05 vs LPS.

To evaluate whether clemastine ameliorated cellular apoptosis *in vivo* and *in vitro*, the apoptosis level was assessed by performing TUNEL assays and western blotting. As shown in Figure 2a, the number of TUNEL-positive cardiomyocytes was higher in CLP-induced rats than in sham-operated rats. Interestingly, clemastine treatment attenuated TUNEL increase in myocardial tissues of septic rats. Consistently, western blotting showed that expression of the pro-apoptotic protein BAX significantly increased and that of the anti-apoptosis protein Bcl-2 significantly decreased in CLP-surgery rats than in sham-operated rats. Interestingly, clemastine significantly reversed these protein activities in the myocardium of septic rats (Figure 2b-d). Consistently, the number of TUNEL-positive nuclei was lesser in LPS+Cle group than in LPS-stimulated group. Moreover, BAX level significantly decreased and Bcl-2 level increased in LPS+Cle cardiomyocytes (Figure 2e-h).

### 3.3 Clemastine attenuates cardiomyocyte mitochondrial damage and dysfunction

**Figure 3.**
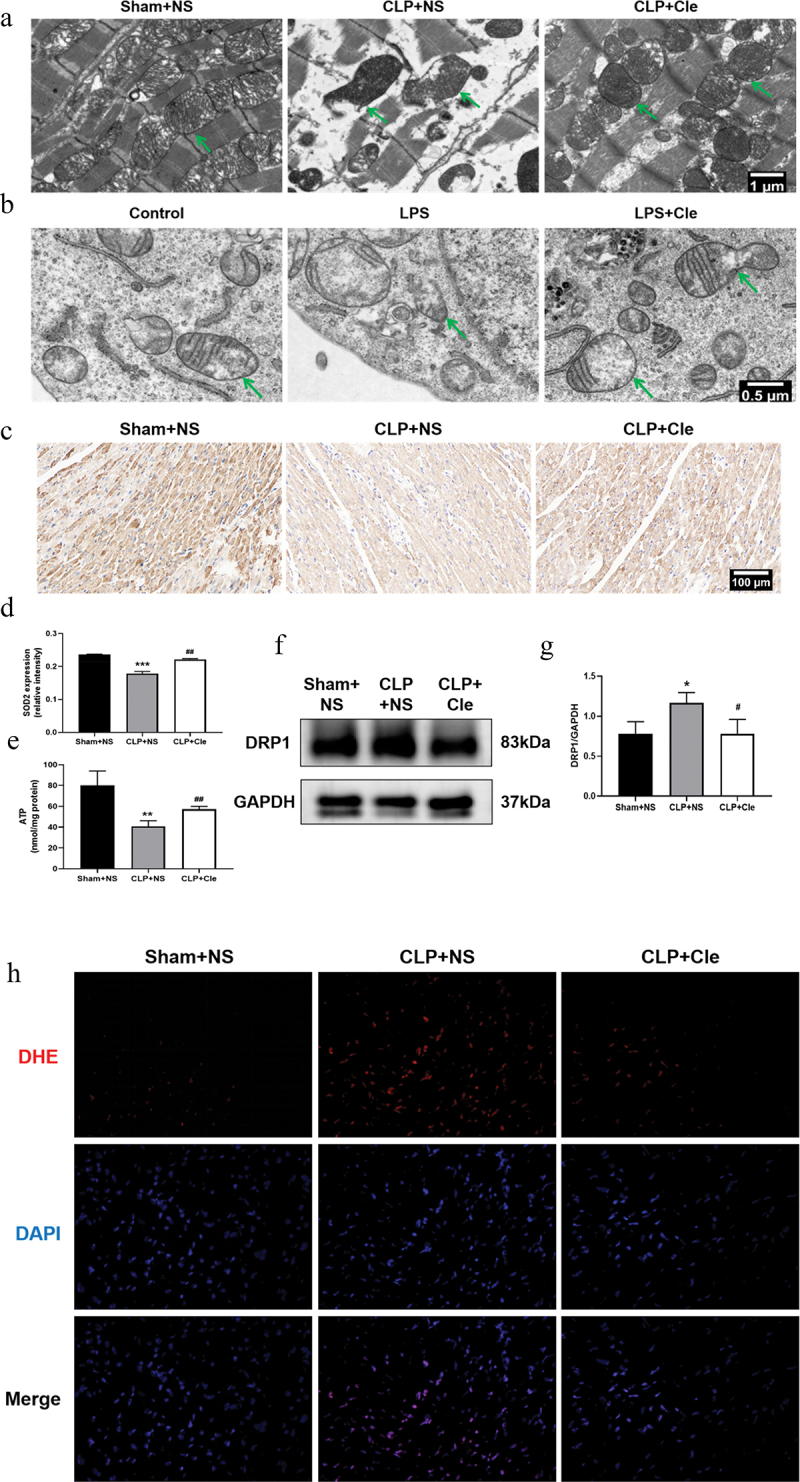
Clemastine attenuates cardiomyocyte mitochondrial damage and dysfunction. (a) TEM showed mitochondrial ultrastructural changes in septic rat hearts, as indicated by green arrows; (b) TEM showed mitochondrial ultrastructural changes in H9c2 cardiomyocytes, as indicated by green arrows (n = 3); (c) Representative myocardial SOD2 immunofluorescence staining images; (d) Relative intensity of myocardial SOD2 expression via immunofluorescence staining; (e) Myocardial ATP levels from respective rat groups; (f) Representative gel blots depicting DRP1 levels *in vivo*; (g) DRP1/GAPDH levels; (h) DHE staining was performed to detect ROS levels in rat heart sections (400X) (n = 3). *P < 0.05 vs Sham+NS, **P < 0.01 vs Sham+NS, ***P < 0.001 vs Sham+NS, ^#^*P* < 0.05 vs CLP+NS, ^##^*P* < 0.01 vs CLP+NS.

Mitochondrial damage and dysfunction dramatically contribute to the deterioration of myocardial function [25]. Therefore, TEM was employed to assess the impact of clemastine administration on mitochondrial ultrastructural changes in septic rats and H9c2 cells. As shown in Figure 3a, TEM revealed swollen mitochondria with broken mitochondrial membranes, disordered iliac crest, ruptured myocardial fibers, and significant vacuolations in CLP+NS group (shown with green arrows). Interestingly, the clemastine group showed decreased mitochondrial swelling and ruptured myocardial fibers, less disordered iliac crest, and a more complete structure. Likewise, TEM revealed broken mitochondrial membranes and disordered iliac crest in the swollen mitochondria of LPS-stimulated H9c2 cells, and clemastine restored mitochondrial ultrastructural abnormalities (Figure 3b).

The amount of ATP, as a source of cardiomyocyte energy, reflects mitochondrial function [26]. The mitochondrial fission protein dynamin-related protein 1 (DRP1) mediates mitochondrial dysfunction during sepsis [27]. Therefore, ATP content and DRP1 expression were examined to evaluate mitochondrial function *in vivo*. As shown in Figure 3e-g, ATP levels significantly decreased in SIMD rats. Interestingly, clemastine administration reversed the changes in ATP levels. Consistently, western blotting revealed that DRP1 expression was significantly upregulated in CLP-surgery rats as compared to that in sham-operated rats. Interestingly, clemastine administration reversed the protein activity in the myocardium of septic rats.

The increase in ROS emissions is considered to be both the cause and effect of damage to the mitochondria in SIMD [28]. Thus, we further detected the ROS levels in rat hearts using DHE dye and mitochondrial antioxidant superoxide dismutase 2 (SOD2) expression. DHE results revealed that ROS levels increased, and immunohistochemistry results showed that SOD2 levels reduced in the CLP group. However, clemastine treatment suppressed ROS overproduction and promoted SOD2 expression (Figure 3c, 3d, 3h).

### 3.4 Clemastine promotes cardiomyocyte autophagy in septic rats and H9c2 cells

**Figure 4.**
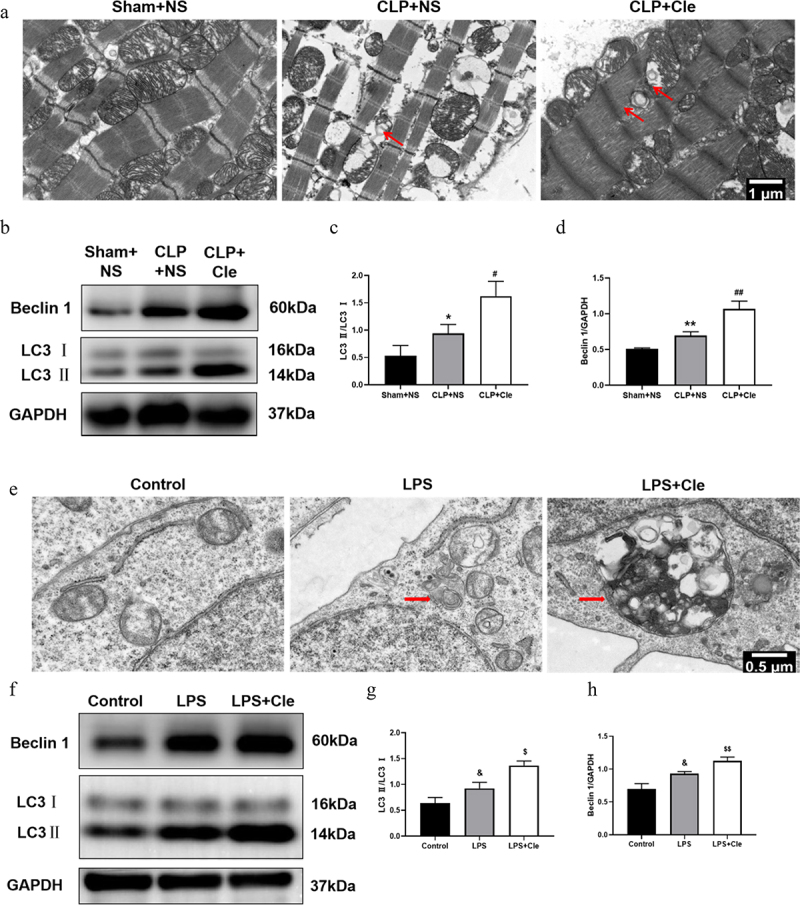
Clemastine promotes cardiomyocyte autophagy in septic rats and H9c2 cells. (a) TEM showing autophagosomes in myocardial tissue,of septic rats as indicated by red arrows; (b) Representative gel blots depicting the levels of autophagy associated proteins Beclin 1 and LC3 *in vivo*; (c) LC3 II/LC3 I levels and (d) Beclin 1/GAPDH levels (n = 3). (e) TEM showing autophagosomes in H9c2 cells; (f) Representative gel blots of Beclin 1 and LC3 *in vitro*; (g) LC3 II/LC3 I levels and (h) Beclin 1/GAPDH levels (n = 3). *P < 0.05 vs Sham+NS, **P < 0.01 vs Sham+NS, ^#^*P* < 0.05 vs CLP+NS, ^##^*P* < 0.01 vs CLP+NS; ^&^*P* < 0.05 vs Control, ^$^*P* < 0.05 vs LPS, ^$$^*P* < 0.01 vs LPS.

TEM was employed to monitor autophagosome changes in rat hearts and H9c2 cells. TEM revealed that a higher number of autophagosomes was observed in the hearts of CLP+NS rats than in those of Sham+NS rats. Interestingly, clemastine treatment after CLP surgery further increased the number of autophagosomes (Figure 4a). Western blotting was performed to evaluate autophagy associated protein expression. As shown in figure 4b-d, Beclin 1 and LC3II/LC3I rate were significantly upregulated in septic rats as compared to that in sham-operated rats. Interestingly, clemastine treatment further increased the expression of LC3II/LC3I and Beclin 1 as compared to that in CLP-induced rats. Likewise, more autophagosomes and increased LC3II/LC3I and Beclin 1 levels were observed in H9c2 cells of Cle+LPS group than in those of LPS group (Figure 4e-h).

### 3.5 Clemastine mitigates myocardial apoptosis via promotion of autophagy

**Figure 5.**
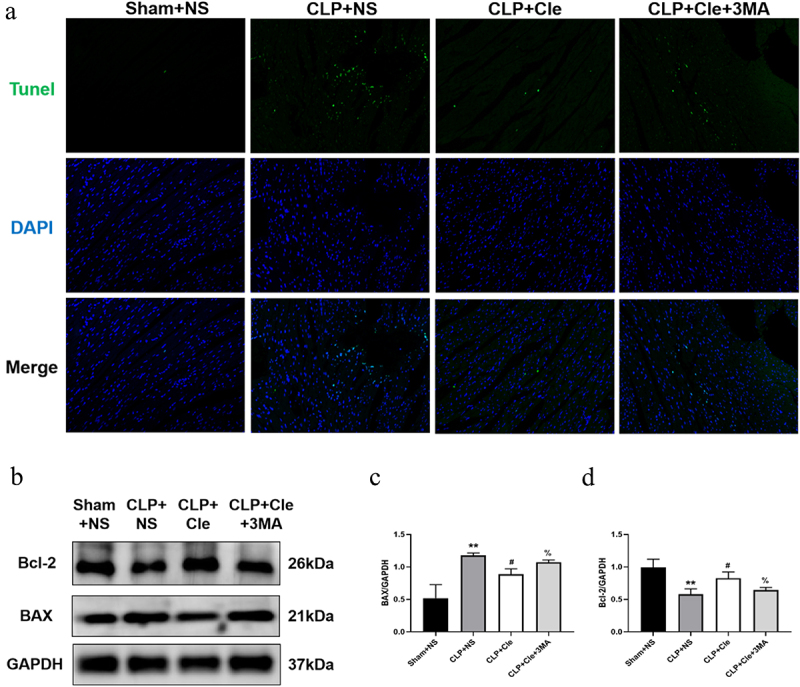
Clemastine mitigates myocardial apoptosis via promotion of autophagy. (a) Representative images depicting TUNEL-positive nuclei from four rat groups (200X); (b) Representative gel blots depicting the levels of BAX and Bcl-2; (c) BAX/GAPDH levels and (d) Bcl-2/GAPDH levels (n = 3). **P < 0.01 vs Sham+NS, ^#^*P* < 0.05 vs CLP+NS, ^%^*P* < 0.05 vs CLP+Cle.

When additional 3-MA was added to the CLP+Cle group, the effect of clemastine weakened. 3-MA intervention increased BAX expression and decreased Bcl-2 expression in the CLP+Cle+3-MA group as compared to that in CLP+Cle group (Figure 5b-d). Further, the results of western blotting were consistent with the immunofluorescence results (Figure 5a), suggesting that clemastine regulated myocardial apoptosis via autophagy; however, the specific mechanisms require further clarification.

## 4. Discussion

In the present study, we found that clemastine ameliorated sepsis-induced myocardial injury *in vivo* and *in vitro*. Clemastine intervention protected against cardiomyocyte apoptosis, mitochondrial damage, and dysfunction, indicating that clemastine is a promising therapeutic target for sepsis-induced cardiac injury. Moreover, our study revealed that clemastine attenuated myocardial apoptosis by promoting autophagy, providing a favorable perspective for sepsis-induced myocardial injury therapy.

Myocardial dysfunction is a common complication of sepsis. Sepsis-induced myocardial injury manifested myocardial contractile function reductions and pathological deterioration of cardiac structures, such as poor LVEF and LVFS, enlarged LVESD, disordered myocardial fibers, and obvious edema. These cardiac structural and functional deteriorations are consistent with the pronounced cellular apoptosis and mitochondrial damage. Induction of apoptosis is responsible for cardiodynamic alterations during sepsis [29]. Studies have shown that myocardial apoptosis is characterized by an abnormal expression of BAX and Bcl-2 [30]. In this study, clemastine effectively reversed the expression of BAX and Bcl-2 and decreased the number of TUNEL-positive nuclei. Our findings are in line with those of a study that revealed myocardial ischemia reperfusion injury prevented by clemastine pre-treatment via cardiac apoptosis regulation [22]. These results suggested that clemastine is an effective agent to resist apoptosis and ameliorate cardiac injury.

Mitochondria occupies approximately 30% of cardiomyocyte volume. In both patients dying of sepsis and CLP-induced mice, changes in the mitochondrial structure, including mitochondrial swelling, cristae rupture, vacuole formation, and internal and external membrane rupture, were observed [15,31]. We observed the same mitochondrial morphological changes in CLP-induced rat hearts and LPS-stimulated H9C2 cells, and clemastine treatment improved the mitochondrial damage. Mitochondrial fusion and fission reach a balance to maintain normal mitochondrial morphology and function under physiological conditions [32]. However, excessive mitochondrial fission mediated by DRP1 causes insufficient ATP supply and subsequent overproduction of ROS during sepsis-induced myocardial injury [27]. In this study, we found that clemastine downregulated the expression of DRP1 and production of ROS, and upregulated ATP levels and antioxidant SOD2 expression. Our results implied that clemastime protected against mitochondrial damage, dysfunction, and mitochondrial oxidative stress.

Autophagy is closely correlated to sepsis, and the autophagy modulation protects against multiple organ injuries such as the heart, liver, and kidney in murine sepsis models [33]. LC3-II and Beclin 1 are common markers of autophagy. LC3-I is modified via ubiquitination and it binds to phosphatidylethoxylamine to form LC3-II, which promotes autophagosome formation [34]. A high LC3II/LC3I ratio suggests the induction of autophagy, and it is positively correlated to formation and activity. Beclin 1 interacts with Vps34 to form a complex that regulates the formation and maturation of autophagosomes [35]. Recent studies have shown that specific overexpression of Beclin 1 can protect myocardial mitochondria and improve cardiac function in a mouse model of LPS-induced sepsis via the pink/parkin pathway [18]. In our study, we found that the LC3II/LC3I ratio and Beclin 1 were significantly elevated *in vivo* and *in vitro* during sepsis. This evidence directly supported autophagy as an adaptive response to stress in pathological conditions. Moreover, higher expression of LC3II/LC3I ratio and Beclin1 was observed following clemastine treatment, suggesting further improvement of autophagy levels. TEM results further confirmed the increase in the number of autophagosomes. Previous studies have found that histamine regulates cardiomyocyte autophagy and apoptosis during acute myocardial infarction via activation of H1R, suggesting that clemastine may affect autophagy and apoptosis through H1R signaling [36]. Autophagy is known to remove damaged organelles, including the mitochondria. However, if autophagy is blocked, excessive unliquidated mitochondria led to a loss of mitochondrial membrane potential, ceventually eliciting cardiomyocyte apoptosis. The current study found that the autophagy inhibitor 3-MA abolished the anti-apoptotic effect of clemastine on CLP-induced myocardial injury, suggesting that clemastine ameliorated myocardial apoptosis in CLP rats via autophagy. Similar to this report, accumulating evidence shows that autophagy upregulation inhibits cardiomyocyte apoptosis triggered via sepsis [16,17]. Therefore, the relationship between autophagy and apoptosis may provide a new treatment method for sepsis-induced myocardial injury.

In conclusion, this study is the first to verify that clemastine protects against mitochondrial structure damage and dysfunction as well as the role of clemastine as an effective agent to prevent sepsis-induced myocardial injury.

This study had several limitations. First, the regulatory element of the effect of clemastine on autophagy requires additional detailed exploration. Second, further investigations are necessary to verify whether clemastine improves mitochondrial dysfunction via autophagy and to elucidate the underlying mechanism of clemastine functioning in sepsis-related mitochondrial dysfunction. Third, further investigations should increase the number of experimental animals to eliminate individual differences.

## 5. Conclusion

Our study demonstrated that clemastine treatment protected against sepsis-induced cardiac dysfunction via mitochondrial protection, apoptosis inhibition, and autophagy improvement both *in vivo* and *in vitro*. Moreover, our results indicated that clemastine ameliorated myocardial apoptosis via autophagy. Therefore, our findings indicate that clemastine is a promising therapeutic strategy for SIMD.

## Data Availability

The datasets used and analyzed during the current study are available from the corresponding author upon reasonable request.
